# Characterization of the Anticholinesterase and Antioxidant Properties of Phytochemicals from *Moringa oleifera* as a Potential Treatment for Alzheimer’s Disease

**DOI:** 10.3390/biomedicines13092148

**Published:** 2025-09-03

**Authors:** Adel M. Aljadaan, Ayman M. AlSaadi, Ibrahim A. Shaikh, Alison Whitby, Arundhati Ray, Dong-Hyun Kim, Wayne G. Carter

**Affiliations:** 1 Department of Pharmacology, College of Pharmacy, Najran University, P.O. Box 1988, Najran 66462, Saudi Arabia; 2 Clinical Toxicology Research Group, School of Medicine, Royal Derby Hospital Centre, University of Nottingham, Derby DE22 3DT, UK; ayman.alsaadi@nottingham.ac.uk (A.M.A.); arundhati.ray@nottingham.ac.uk (A.R.); 3 Children’s Brain Tumour Research Centre, School of Medicine, Biodiscovery Institute, University of Nottingham, Nottingham NG7 2RD, UK; alison.whitby@nottingham.ac.uk; 4 Centre for Analytical Bioscience, Advanced Materials and Healthcare Technologies Division, School of Pharmacy, University of Nottingham, Nottingham NG7 2RD, UK; dong-hyun.kim@nottingham.ac.uk; 5 College of Pharmacy, Kyungpook National University, Daegu 41566, Republic of Korea

**Keywords:** Alzheimer’s disease, anticholinesterase, antioxidant, 4-*O*-caffeoylquinic acid, chlorogenic acid, *Moringa oleifera*, neurodegenerative diseases, quercetin 3-β-D-glucoside, rutin

## Abstract

**Background/Objectives:** Alzheimer’s disease (AD) is the most prevalent form of dementia and is characterized by a decline in cognition that may be due, in part, to deficient cholinergic signalling. Cholinesterase inhibitors (ChEIs) are the first-line pharmacotherapies for treating the diminished cholinergic function in AD patients. Plant phytochemicals may provide useful ChEIs and mitigate other elements of AD pathology, including oxidative stress. **Methods**: Herein, the phytochemicals present in *Moringa oleifera* aqueous and methanolic extracts were identified by LC-MS/MS and the potential of several phytochemicals (4-*O*-caffeoylquinic acid (4-CQA), quercetin 3-β-D-glucoside (Q3-β-D), chlorogenic acid (CGA), and rutin) to act as ChEIs and antioxidants was assessed. **Results**: The phytochemicals inhibited human acetylcholinesterase (AChE) in the following order of potency: 4-CQA > Q3-β-D > CGA > rutin; for AChE from *Electrophorus electricus*, the order of potency was Q3-β-D > 4-CQA > CGA > rutin. For human butyrylcholinesterase (hBuChE), the order of potency was rutin > 4-CQA > Q3-β-D > CGA and for equine serum BuChE, it was 4-CQA > Q3-β-D > rutin > CGA. Molecular docking validated the binding of the phytochemicals to cholinesterases, with binding affinities comparable to or higher than those of ChEI drugs. All the phytochemicals displayed potent radical-scavenging and antioxidant activities across six assays. 4-CQA was the most effective antioxidant in three of the assays. **Conclusions**: *M. oleifera* contains phytochemicals with weak ChEI activity and potent antioxidant capacity, with potential use as nutraceuticals to treat the cholinergic signalling deficit and oxidative stress that typifies AD pathology.

## 1. Introduction

Dementia is a leading cause of morbidity and mortality among the elderly. It poses a substantive global health challenge, with the number of people living with dementia estimated to rise to 78 million by 2030 [1]. Alzheimer’s disease (AD) is the most common form of dementia, accounting for 60‒70% of cases [2]. AD is an irreversible neurodegenerative disease with symptoms that include depression, cognitive impairment, confusion, and memory loss. These symptoms typically arise after the age of 65 and become progressively worse, resulting in premature death [3,4,5]. AD has an idiopathic aetiology, although several risk factors in addition to ageing can confer an increased relative risk of disease development. These include genetic predisposition, including mutations in the apolipoprotein E (*APOE*) gene [6,7], and exposure to environmental toxins such as pesticides [8].

At autopsy, patients with AD typically display atrophy in the entorhinal cortex and hippocampus, consistent with the reduced functionality of these brain regions and the associated symptomology (memory impairment) displayed by patients [9]. Based on post-mortem analyses, the key histopathological features of AD include the deposition and accumulation of extracellular amyloid-beta (Aβ) and intracellular (hyperphosphorylated) tau proteins, leading to intracellular neurofibrillary tangles (NFTs). These may show neurotoxic prion-like spreading within brain tissues [9,10,11]. AD, like other neurodegenerative diseases, is also pathologically influenced by neuroinflammation and the production of excessive reactive oxygen species (ROS) and the associated redox stress [12,13,14]. Cellular oxidative stress arises when reactive free radicals overwhelm the cell’s ability to dissipate them using endogenous enzymatic and non-enzymatic antioxidants [12,13,14].

Current AD treatments are limited to managing symptoms and slowing disease progression. The approved pharmacotherapies for AD are cholinesterase inhibitors (ChEIs) (donepezil, galantamine, and rivastigmine) and memantine, an N-methyl-D-aspartate (NMDA) receptor antagonist. The rationale for the use of ChEIs is to restore the acetylcholine signalling deficit (by sustaining acetylcholine levels) in AD patients and thereby increase cholinergic neurotransmission. Memantine attenuates the pathological NMDA receptor excitotoxicity in AD caused by elevated levels of glutamate [15,16]. Although other therapies have been developed, including biological therapies such as anti-amyloid monoclonal antibodies [15,16], pharmacotherapy remains the mainstay, cost-effective treatment. However, neither ChEIs nor memantine are efficacious in all patients and they can elicit undesired side effects [15]. Therefore, it is imperative to develop new ChEIs and compounds that are able to counter other elements of AD pathophysiology, including cellular redox stress.

Plants and traditional remedies and medicines are still widely used, particularly in some low- to middle-income countries [17]. *Moringa oleifera*, from the Moringaceae family, is known as the ‘miracle tree’, and is widely distributed in Asia and Africa and exploited for its plant extracts and phytochemicals with broad pharmacological properties and ethnomedicinal health benefits [18,19,20]. Previously, we have shown that plant extracts from *M. oleifera* leaves display concentration-dependent antioxidant and potent ChEI activities in vitro [21], consistent with the neuroprotective and cognitive-enhancing abilities reported in vivo [22,23,24,25]. Collectively, these studies and others suggest that *M. oleifera* could counter the cholinergic deficit associated with AD and other neurodegenerative diseases [26] due to its high levels of bioactive compounds [17,26].

In this study, to characterize the potentially bioactive constituents of *M. oleifera*, the phytochemicals in aqueous and ethanolic extracts of *M. oleifera* were identified. Subsequently, the in vitro ChEI activity, binding affinity to cholinesterases (using molecular modelling), and antioxidant capabilities of several phytochemicals were characterized.

## 2. Materials and Methods

### 2.1. Reagents and Chemicals

Unless specified otherwise, all chemicals were purchased from Sigma (Poole, UK), including the phytochemicals, 4-*O*-caffeoylquinic acid (product 65969), quercetin 3-β-D-glucoside (product 17793), chlorogenic acid (product PHR2202), and rutin (product R5143).

### 2.2. Liquid Chromatography–Mass Spectrometry Analysis

Ethanolic and aqueous extracts of *M. oleifera* were produced according to the methods of a previous publication [27]. The extracts were diluted in methanol at concentrations of 10–300 mg/mL before being separated using a Dionex UltiMate 3000 high-performance liquid chromatography (HPLC) system coupled to a Q-Exactive Plus hybrid quadrupole-orbitrap mass spectrometer with a heated electrospray ionization (HESI) source (Thermo Fisher Scientific, Hemel Hempstead, UK). Liquid chromatography–mass spectrometry (LC-MS) was performed as described in a previous publication [27].

### 2.3. Assessment of Cholinesterase Inhibitory Activity

The ability of agents to inhibit the activity of human acetylcholinesterase (hAChE) (C1682, Sigma, Irvine, UK), human butyrylcholinesterase (hBuChE) (B4186, Sigma, Irvine, UK), acetylcholinesterase from *Electrophorus electricus* (electric eel) (eeAChE) (C3389, Sigma, Irvine, UK), and butyrylcholinesterase from horse (equine) serum (esBuChE) (C7512, Sigma, Irvine, UK) were measured using a modified version of Ellman’s method [28] that was adapted to be read with a 96-well microplate reader (VersaMax Molecular Devices, San Jose, CA, USA) [29]. A 5 min kinetic assay was performed with each phytochemical using 3 µL of either hAChE or eeAChE for a final enzyme concentration of 0.44 U/mL in a total volume of 200 µL (diluted with 0.1 M phosphate-buffered saline (PBS)). For hBuChE and esBuChE, a final concentration of 0.5 U/mL was used in each assay due to the slower kinetic response of BuChE during the first five minutes of the reaction compared to AChE. The assays were conducted in a final volume of 200 µL, which consisted of 150 µL of 0.01 M 5,5-dithio-bis(2-nitrobenzoic) acid (DTNB), 46 µL of 0.1 M PBS or inhibitor, and 4 µL of substrate (either acetylthiocholine iodide (ATCI) for AChE or butyrylthiocholine iodide (BTCI) for BuChE). Following incubation with phytochemicals or positive control ChEIs, the resulting thiocholine reacted with 5,5-dithiobis-(2-nitrobenzoate) ions to form yellow 5-thio-2-nitrobenzoate (TNB2) anions. This product was quantified using visible spectroscopy to assess ChE inhibition, with absorbance readings measured at 412 nm. The phytochemicals were tested at 75, 100, 500, 750, and 1000 µM concentrations. ChE inhibition was assessed after a pre-incubation of 20 min at room temperature protected from light using aluminium foil. The enzymatic reaction was initiated by adding 4 µL of substrate using a multi-channel automated pipette, before readings were taken every 30 s for 5 min using the kinetic assay of a Multiskan Spectrum (Thermo Electron Corporation, Vantaa, Finland). Nonlinear regression was performed using GraphPad Prism V.10 (San Diego, CA, USA; https://www.graphpad.com/scientific-software/prism/) to calculate the actual or theoretical concentration of inhibitors that inhibited cholinesterase activity by 50% (IC_50_).

### 2.4. In Silico Molecular Docking

Molecular modelling investigations were conducted using MOE 2015.10, a software provided by Chemical Computing Group Inc. (Montreal, QC, Canada). In silico molecular docking experiments were conducted to investigate the interactions of human acetylcholinesterase (hAChE) and butyrylcholinesterase (hBuChE) with selected phytochemical ligands. Energy-minimized ligands were superimposed onto the co-crystallized structures of donepezil (E20) and N-(1-(2,3-dihydro-1H-inden-2-yl)piperidin-3-yl)methyl-N-(2-(dimethylamino)ethyl)-2-naphthamide (92H), as represented by the Protein Data Bank (PDB) entries 7E3H and 5NN0, respectively (https://www.rcsb.org/) The co-crystallized ligands were removed prior to docking. Molecular docking was performed using MOE 2015.10, the default Triangle Matcher placement method, and the GBVI/WSA ∆G scoring function to estimate the free energy of ligand binding. Gasteiger–Hückel charges were applied, and water molecules were excluded from the docking simulations. The 3D structures of the ligands were generated and energy-minimized within MOE using its integrated force field. Ligand–enzyme complexes exhibiting the lowest binding energy scores, indicative of the highest binding affinities, were selected for further analysis.

### 2.5. Antioxidant Assays

#### 2.5.1. 2,2-Diphenyl-1-picrylhydrazyl (DPPH) Free Radical-Scavenging Assay

The 2,2-diphenyl-1-picrylhydrazyl radical (DPPH•)-scavenging assay, which measures the relative ability of an antioxidant to neutralize the DPPH radical, was performed according to the method of a previous publication [30]. The DPPH radical’s unpaired electron absorbs strongly at 517 nm. Phytochemicals (20 µL) at final concentrations of 10, 50, 75, 500, 750, and 1000 µM and 180 µL of 0.1 mM DPPH (prepared in ethanol) were added into a 96-well microtiter plate. The negative control consisted of 20 µL of ultrapure water and the positive controls were the known antioxidants gallic acid and α-tocopherol (vitamin E). The samples were incubated at 37 °C for 40 min protected from the light using aluminium foil, and then the remaining DPPH radicals were quantified by spectrophotometry at 517 nm (Multiskan Spectrum, Thermo Electron Corporation, Vantaa, Finland) as an endpoint measurement. All samples were examined in triplicate. The negative control adsorption values were subtracted from the measured values. The percentage inhibition of DPPH was calculated using the following equation:

DPPH• scavenging (%) = 100 × (A_0_ − A_1_)/A_0_

where (A_0_) denotes the absorbance of the control reaction and (A_1_) denotes the absorbance of the reactions with the phytochemicals.

#### 2.5.2. Ferric Reducing Antioxidant Power (FRAP) Assay

The potential of phytochemicals to reduce ferric (Fe^3+^) ions to ferrous (Fe^2+^) ions was used as a measure of the relative reducing (antioxidant) capacity using the method of a previous publication [30]. The phytochemicals were tested at final concentrations of 10, 50, 75, 500, 750, and 1000 µM. To 400 µL of PBS, 4 µL of each of the phytochemicals or controls was added and then this was mixed with 250 µL of 1% potassium ferricyanide, and then the sample was incubated at 50 °C for 20 min, before 250 µL of 10% (*w*/*v*) trichloroacetic acid was added and the mixture was thoroughly vortexed. The samples were then centrifugated for 10 min at 3000 rpm. A 100 µL volume of the supernatant was removed and transferred into a 96-well microtiter plate. A 100 µL volume of ultrapure water was added along with 20 µL of a freshly prepared 0.1% ferric chloride solution. The level of Perl’s Prussian blue produced in the reaction was read at 700 nm using a Varioskan™ LUX multimode microplate reader (ThermoFisher, Stafford, UK), and the antioxidant ability was compared with that of gallic acid and L-ascorbic acid.

#### 2.5.3. Lipid Peroxidation Inhibition (LPI) Assay

The ability of the phytochemicals to inhibit lipid peroxidation was measured according to the method of ALNasser et al. (2022) [30]. A 100 µL volume of bovine brain extract type I (% mg/mL), Folch fraction I (Sigma, B1502), was mixed with 100 µL of the phytochemicals (final concentrations of 10, 50, 75, 500, 750, and 1000 µM), 40 µL of ultrapure water, and 30 µL of PBS, which were then combined with 100 µL of 5 mM sodium nitroprusside as the pro-oxidant. Following that, the samples were incubated at 37 °C for 2 h and then 500 µL of acetic acid, 300 µL of 8.1% sodium dodecyl sulphate, and 500 µL of 0.8% thiobarbituric acid were added to the mixture. This mixture was subsequently incubated at 85 °C for 45 min to facilitate the formation of a coloured malondialdehyde (MDA) product. After the incubation, the samples were cooled, and then 200 µL from each sample was transferred into a 96-well microtiter plate and the absorbance of the coloured MDA product was measured at 532 nm using a VarioskanTM LUX multimode microplate reader (ThermoFisher, Stafford, UK). Gallic acid and L-ascorbic acid were similarly processed to provide a reference as known antioxidants. The percentage of LPI was determined using the same formula that was used to calculate the DPPH free radical-scavenging activity.

#### 2.5.4. Hydroxyl Radical-Scavenging Assay

The ability of the phytochemicals to scavenge the hydroxyl radical (•OH) produced by a Fenton reaction using an Fe^3+^–ascorbate–ethylenediaminetetraacetic acid–H_2_O_2_ system was performed according to the method of a previous publication [30]. The reaction mixture had a final volume of 200 µL, which consisted of 50 µL of 2-deoxy-2-ribose sugar (12 mM), 20 µL of fresh ferric chloride (FeCl_3_) (1 mM), 20 µL of ethylenediaminetetraacetic acid (1 mM), 30 µL of PBS, 50 µL of H_2_O_2_ (8 mM), 20 µL of L-ascorbic acid (1 mM), and 10 µL of the phytochemical at concentrations of 10, 50, 75, 500, 750, and 1000 µM. After incubating for 45 min at 37 °C, the reaction mixture was supplemented with 40 µL of a mixture of 2-thiobarbituric acid (TBA) (0.5% in 0.025 M sodium hydroxide solution) and 2.8% trichloroacetic acid, and then the mixture was incubated for 15 min at 85 °C to generate a pink chromogen. After cooling, 200 µL of this solution was transferred into a 96-well microtiter plate, and the absorbance was read at 532 nm using a VarioskanTM LUX multimode microplate reader (ThermoFisher, Stafford, UK). Gallic acid and L-ascorbic acid were similarly processed and used as reference antioxidant standards. The percentage of inhibition and free radical-scavenging activity was calculated according to the equation used to calculate the DPPH radical-scavenging activity.

#### 2.5.5. 2,2′-Azino-bis(3-ethylbenzthiazoline-6-sulfonic acid) (ABTS) Radical-Scavenging Assay

An assessment of the relative ability of the phytochemicals to scavenge the ABTS radical cation (ABTS•^+^) was undertaken using a spectrophotometric assay according to the method of a previous publication [30]. Potassium persulfate (2.45 mM) and ABTS (7 mM) solutions were prepared in ultrapure water and then a working solution was generated by combining 3 mL of each solution and incubating the reaction for 12–16 h at room temperature (25 °C) in the dark. The solution was then diluted by combining 1 mL of the ABTS radical solution with 25 mL of PBS to achieve an absorbance of 0.70 at 750 nm, which was measured using a VarioskanTM LUX multimode microplate reader (ThermoFisher, Stafford, UK). A 190 μL volume of this radical solution was added to a well of a 96-well microtiter plate and then 10 μL of a phytochemical was added to achieve final concentrations of 10, 50, 75, 100, 500, 750, and 1000 μM. After mixing, the plate was incubated for 5 min in the dark, and then the absorbance was read at 750 nm using a VarioskanTM LUX multimode microplate reader (ThermoFisher, Stafford, UK). The phytochemical activity was assessed relative to the activity of antioxidant standards: gallic acid, α-tocopherol, and L-ascorbic acid. The percentage of ABTS•^+^ free radical-scavenging activity was calculated using the equation used to calculate the DPPH radical-scavenging activity.

#### 2.5.6. Nitric Oxide (NO) Radical-Scavenging Assay

The ability of the phytochemicals to scavenge nitric oxide radicals (•NO) was measured using a previously published protocol [30]. A 2 mL volume of a 10 mM sodium nitroprusside solution (prepared in PBS) was mixed with 0.5 mL of the phytochemicals (final concentrations of 10, 50, 75, 100, 500, 750, and 1000 μM) or butylated hydroxyanisole (BHA) at concentrations of 0.1–500 µg/mL. After an incubation period of 2.5 h at 25 °C, 0.5 mL of the solution was mixed with an equivalent volume of Griess reagent (1 mL of 0.33% sulphanilamide dissolved in 20% glacial acetic acid) and 1 mL of 0.1% (*w*/*v*) N-(1-naphthyl)ethylenediamine dihydrochloride and incubated for 5 min at room temperature. The reaction was then allowed to proceed for 30 min at room temperature before 200 µL was dispensed into a 96-well microtiter plate, and the absorbance was measured at 540 nm using a VarioskanTM LUX multimode microplate reader (Thermo Fisher, Stafford, UK). The phytochemical activity was assessed relative to the activity of antioxidant standards (gallic acid, α-tocopherol, and L-ascorbic acid) with the percentage of •NO inhibition calculated using the equation used to calculate the DPPH radical-scavenging activity.

### 2.6. Statistical Analysis

The samples were tested in triplicate and the mean ± standard error was plotted using GraphPad PRISM v10 (GraphPad Software Inc., San Diego, CA, USA. www.graphpad.com). The concentrations of the phytochemicals or drugs that produced either 50% inhibition (IC_50_) or 50% effective concentration (EC_50_) were calculated using non-linear regression and PRISM. A one-way analysis of variance with Tukey’s multiple comparisons post-test was conducted using PRISM and a *p*-value below 0.05 was defined as statistically significant.

## 3. Results

### 3.1. Liquid Chromatography–Mass Spectrometry (LC-MS)

Analysis of the aqueous (phosphate-buffered saline (PBS)) and ethanolic extracts of *M. oleifera* using LC-MS/MS revealed 47 major phytochemicals, including phenolic compounds, flavonoids, as well as fatty acids and amino acids (Table 1). There were differences between the aqueous and ethanolic extracts, with a higher recovery of phenolic compounds, phenolic acids, and amino acids in the aqueous extract, and a higher recovery of flavonoid and fatty acids in the ethanolic extract. Some of the identified phytochemicals have well-characterized antioxidant activities (see Table 1).

**Figure 1:**
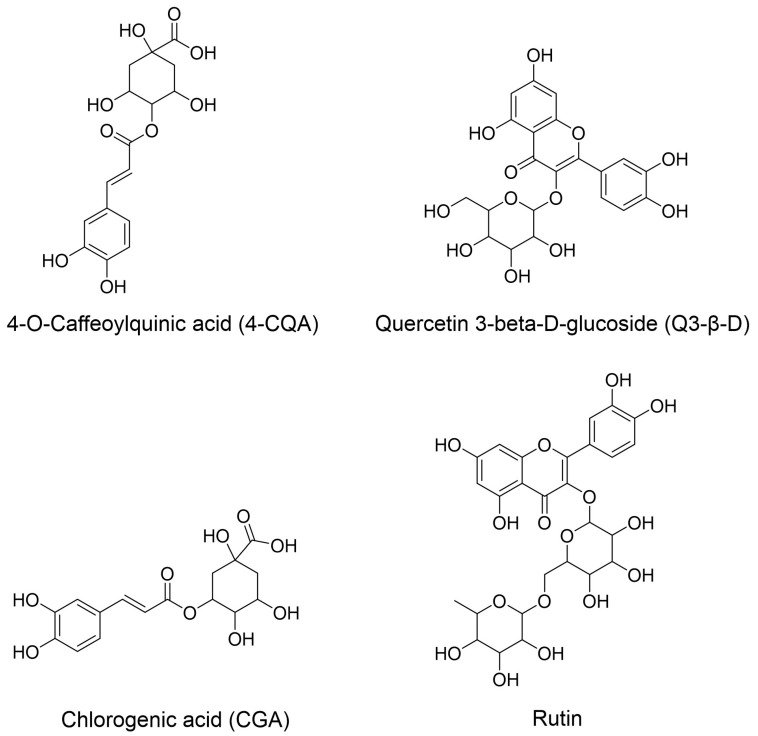
Structures of the analysed phytochemicals. The structures were taken from PubChem and drawn using ChemDraw (version 23.1.1.3).

A number of the phytochemicals that were detected in the *M. oleifera* extracts were also previously reported to be present within plant extracts that demonstrated the ability to halt cognitive decline [51]. Furthermore, an in silico analysis of several of these phytochemicals (methyl 4-caffeoylquinate, 3-caffeoylquinic acid, quercetin 3-*O*-glucoside, and quercetin 3-rutinoside (rutin)) indicated that they may possess ChEI activity [51]. This prompted us to assess the ChEI activity and antioxidant properties of these chemicals individually, in vitro, with 4-*O*-caffeoylquinic acid used as the commercially available compound closest to methyl 4-caffeoylquinate. The structures of the analysed compounds are shown in Figure 1.

### 3.2. In Vitro Cholinesterase Inhibition by Phytochemicals

**Figure 2:**
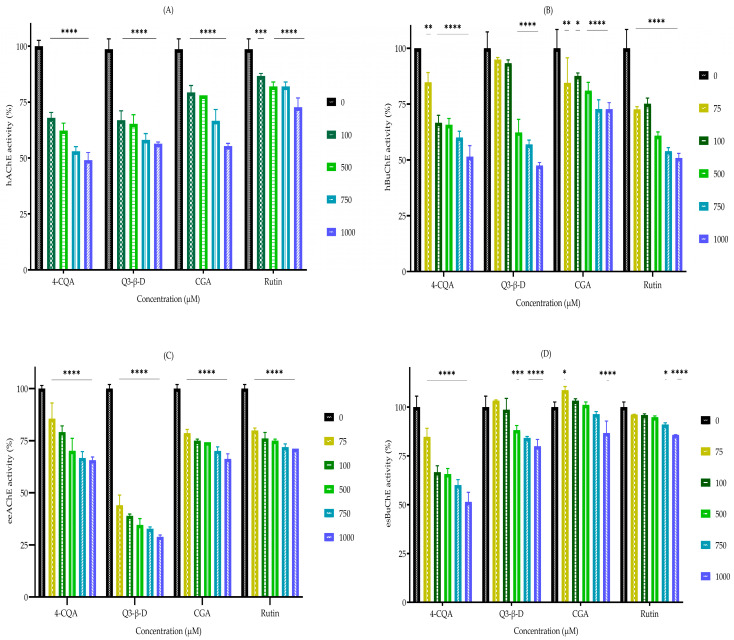
Assessment of cholinesterase inhibition by phytochemicals. Phytochemical inhibition of cholinesterases (**A**) hAChE, (**B**) hBuChE, (**C**) eeAChE, and (**D**) esBuChE were quantified using a modified version of Ellman’s assay. Histograms represent the mean ± SEM from at least three replicate assays for each phytochemical concentration (n = 3). * *p* < 0.05, ** *p* < 0.01, *** *p* < 0.001, and **** *p* < 0.0001. Abbreviations: CGA, chlorogenic acid; 4-CQA, 4-*O*-caffeoylquinic acid; eeAChE, *Electrophorus electricus* acetylcholinesterase; esBuChE, equine serum butyrylcholinesterase; hAChE, human acetylcholinesterase; hBuChE, human butyrylcholinesterase; Q3-β-D, quercetin 3-β-D-glucoside; CGA, chlorogenic acid.

The phytochemicals were tested for their ability to inhibit AChE and BuChE using a modified version of Ellman’s assay [28]. The selected phytochemicals 4-*O*-caffeoylquinic acid (4-CQA), quercetin 3-beta-D-glucoside (Q3-β-D), chlorogenic acid (CGA), and rutin all displayed broad inhibitory effects on human acetylcholinesterase (hAChE) and human butyrylcholinesterase (hBuChE), with more potent inhibition against hAChE. Similarly, the AChE from *Electrophorus electricus* (eeAChE) was more potently inhibited than equine serum butyrylcholinesterase (esBuChE) (Figure 2). 4-CQA was the most potent inhibitor of hAChE, hBuChE, and esBuChE; Q3-β-D was the most potent inhibitor of eeAChE; rutin was the weakest hAChE inhibitor but the most potent hBuChE inhibitor, as shown in Figure 2 and Table 2.

The inhibitor (phytochemical) concentration that reduced acetyl- or butyrylcholinesterase activity by 50% (actual or estimated) was calculated using non-linear regression; the values are shown in Table 2. All the phytochemicals were relatively weak ChEIs when compared with the Food and Drug Administration (FDA)-approved ChEIs (rivastigmine, donepezil, and galantamine) assayed under the same conditions (Table 2 and Supplementary Figure S1). The commercial ChEI drugs were all potent AChE inhibitors with relatively low IC_50_ values (Table 2) and rivastigmine was also a potent BuChE inhibitor. Eserine (physostigmine), a recognized AChE inhibitor [29], was used as a positive control and exhibited potent AChE inhibitory activity; likewise, ethopropazine, a documented inhibitor of BuChE [52], was also used as a positive control, displaying potent BuChE inhibitory activity under the experimental conditions (Table 2).

### 3.3. In Silico Phytochemical Docking to Cholinesterases

**Figure 3:**
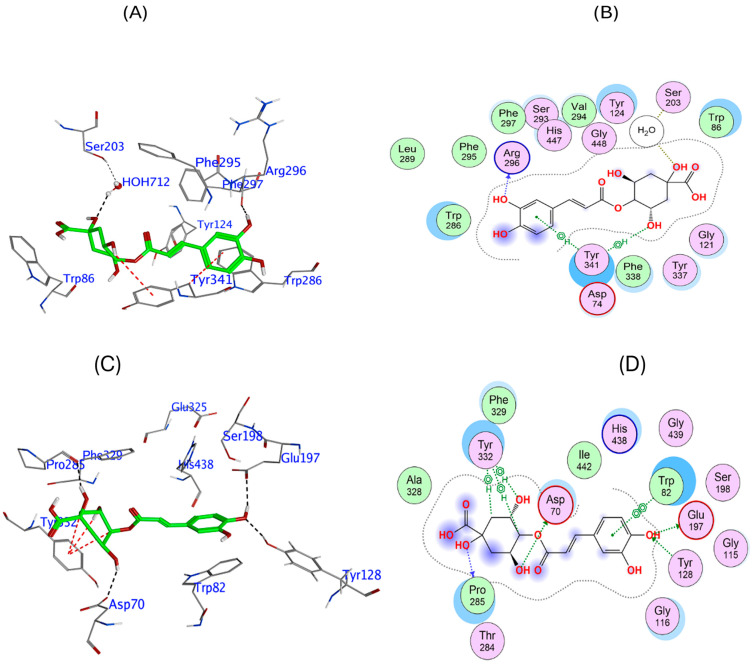
Molecular interactions between 4-*O*-caffeoylquinic acid and hAChE and hBuChE. In silico model of the association of 4-*O*-caffeoylquinic acid with human AChE shown as a 3D representation of the interactions (**A**) and a 2D depiction of the molecular interactions (**B**). Association of 4-*O*-caffeoylquinic acid with human BuChE shown as a 3D representation of the interactions (**C**) and 2D depiction of the molecular interactions (**D**).

To provide further insight into the potential of the phytochemicals to act as cholinesterase inhibitors, molecular in silico docking was conducted. This docking analysis provided a guide to compound orientation at the enzymatic active site, which for hAChE, showed that 4-CQA is predicted to form strong interactions within the enzyme’s catalytic and anionic sites, forming hydrogen bonds with Arg296 and further stabilizing its position via a water molecule (HOH 712) and π interactions with Tyr341 (Figure 3A,B). These interactions spanned critical regions within the active site, potentially obstructing substrate access and thereby decreasing catalytic efficiency, with a relatively potent binding affinity of −9.12 kcal/mol (Table 3). The docking results for 4-CQA phytochemical ligand binding to hBuChE revealed a modest predicted binding strength, with interactions at the enzyme’s active site, including several hydrogen bonds with key active site residues, including Glu197 and Asp70 near the catalytic triad, as well as Pro285 and Tyr128. Additional π interactions with Tyr332 contributed to its binding energy of −7.97 kcal/mol (Figure 3C,D).

**Figure 4:**
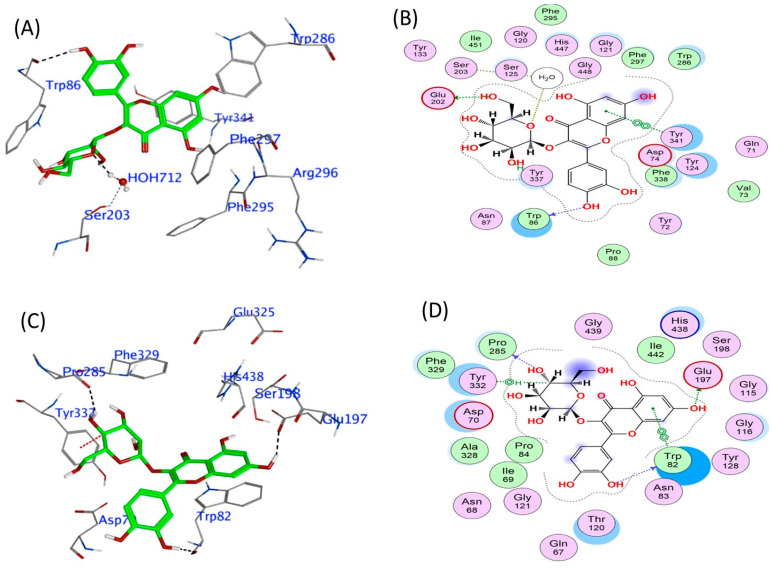
Molecular interactions between quercetin 3-β-D-glucoside and hAChE and hBuChE. In silico modelling of the association of quercetin 3-β-D-glucoside with human AChE shown as a 3D representation of the interactions (**A**) and 2D depiction of the molecular interactions (**B**). Association of quercetin 3-β-D-glucoside with human BuChE shown as a 3D representation of the interactions (**C**) and 2D depiction of the molecular interactions (**D**).

The model of Q3-β-D binding to human AChE shows that it binds to critical residues within the active site, forming hydrogen bonds with Glu202 and π-π stacking interactions with Tyr341 and Trp86. This binding effectively obstructs the access of acetylcholine to the catalytic site, thereby inhibiting its binding and hydrolysis. Such interactions could result in reduced cholinesterase enzymatic activity. Furthermore, π-π stacking interactions with Trp86 would enhance its stable binding conformation, which may hinder substrate access (Figure 4A,B), and contribute to the relatively stable predicted binding affinity of −9.96 kcal/mol (Table 3). Similarly, Q3-β-D had a strong predicted binding affinity of −10.27 kcal/mol (refer to Table 3) for human BuChE, forming hydrogen bonds with Trp82, Glu197, and Pro285. The orientation of Q3-β-D also facilitates direct engagement with catalytic residues and stabilized positioning through a π interaction with Tyr332 (Figure 4C,D).

**Figure 5:**
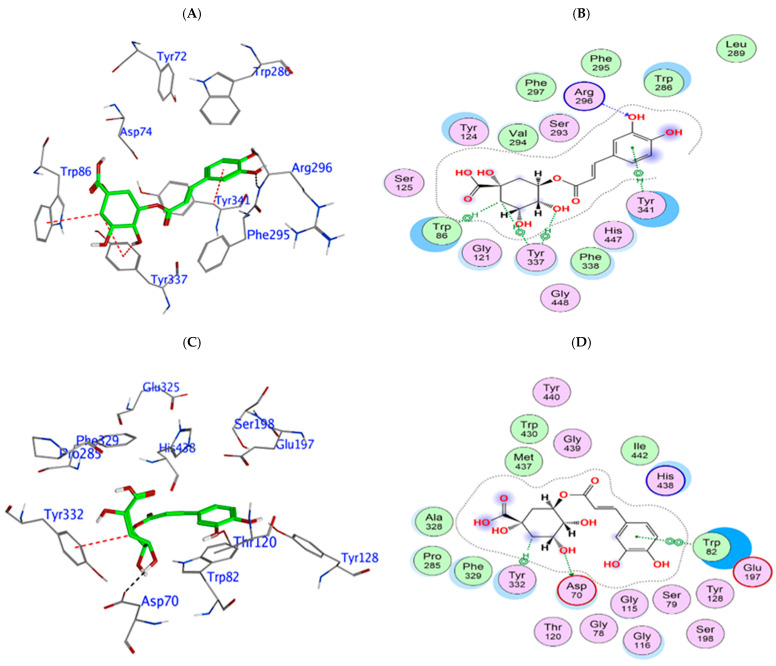
Molecular interactions between chlorogenic acid and hAChE and hBuChE. In silico modelling of the association of chlorogenic acid with human AChE shown as a 3D representation of the interactions (**A**) and 2D depiction of the molecular interactions (**B**). Association of chlorogenic acid with human BuChE shown as a3D representation of the interactions (**C**) and 2D depiction of the molecular interactions (**D**).

The binding energy of chlorogenic acid binding to human AChE was relatively strong and predicted to be −9.69 kcal/mol (Table 3); the binding occurs through interactions such as hydrogen bonding with Arg296 and H-π interactions with Trp86 and Tyr337 (Figure 5A,B). Its positioning within the cholinergic binding sites suggests that it could potentially disrupt the enzyme’s catalytic activity. Chlorogenic acid showed a similar and relatively strong binding profile for human BuChE (−9.69 kcal/mol, Table 3), arising from an association with Trp82 and Tyr332 (Figure 5C,D).

**Figure 6:**
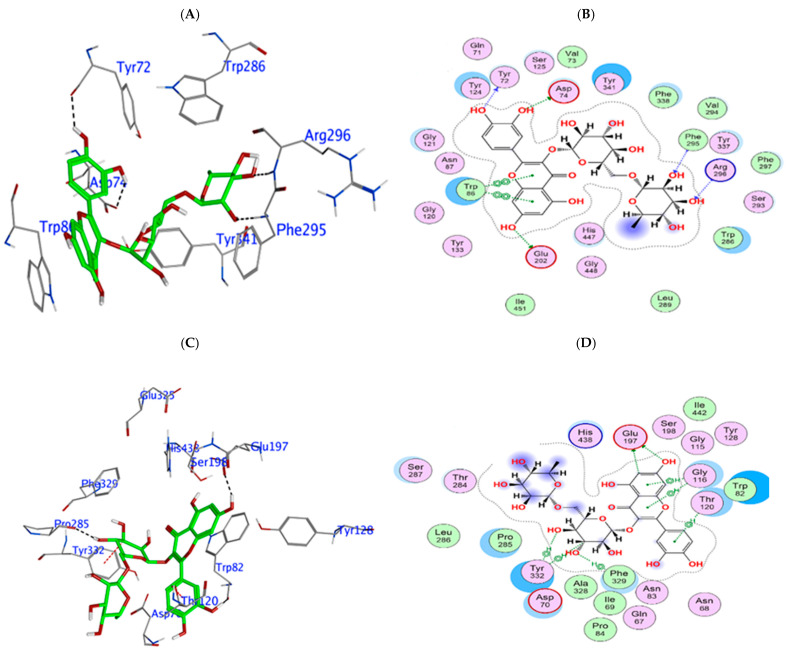
Molecular interactions between rutin and hAChE and hBuChE. In silico modelling of the association of rutin with human AChE shown as a 3D representation of the interactions (**A**) and 2D depiction of the molecular interactions (**B**). Association of rutin with human BuChE shown as a 3D representation of the interactions (**C**) and 2D depiction of the molecular interactions (**D**).

Rutin had the strongest predicted binding strength for human AChE at −14.81 kcal/mol (Table 3), with an expansive interaction profile involving multiple hydrogen bonds with catalytic residues (Asp74 and Glu202) and gating residues (Tyr72 and Arg296) (Figure 6A,B). Similarly, rutin had the highest binding affinity for hBuChE at −12.03 kcal/mol (Table 3), with an extensive interaction profile that included hydrogen bonds with Asn68, Asp70, Trp82, Glu197, and Pro285. The interaction of rutin with Tyr332 suggests additional π-π stacking, further reinforcing its fit within the active site (Figure 6C,D).

A comparative analysis of the docking interactions of 4-CQA, Q3-β-D, CGA, and rutin with hAChE and hBuChE revealed several key patterns. 4-CQA exhibited substantial interactions with both enzymes but had a stronger binding affinity for hAChE, where it engaged Arg296 and Tyr341 in the active site. With hBuChE, the interactions involved Glu197, Asp70, and other peripheral residues, which could reflect a different inhibitory mechanism. Q3-β-D demonstrated strong binding affinities for both enzymes, utilizing hydrogen bonds with essential catalytic residues such as Glu202 in hAChE and Glu197 in hBuChE. It also had additional π-π stacking and interactions with Tyr337 and Tyr332. This indicates that Q3-β-D could be an effective dual inhibitor. CGA showed similar interaction profiles with both enzymes, and its positioning in both active sites likely interfere with catalytic activity, though its inhibitory effect may be comparatively moderate. Interestingly, rutin emerged as the most potent inhibitor for both hAChE and hBuChE. Rutin displayed a broad interaction profile with both enzymes, which included hydrogen bonds with critical catalytic and peripheral residues, along with π-π stacking interactions, which result in a high likelihood for obstruction of substrate access and the formation of a stable binding conformation in the active sites of both enzymes.

### 3.4. Radical-Scavenging and Antioxidant Properties of the Phytochemicals

The phytochemicals 4-CQA, Q3-β-D, CGA, and rutin were evaluated for their relative radical-scavenging activities and antioxidant properties using a panel of assays, which were compared with the activities of known antioxidants: gallic acid, ascorbic acid (vitamin C), and α-tocopherol (vitamin E).

#### 3.4.1. 2,2-Diphenyl-1-picrylhydrazyl Radical-Scavenging Assay

**Figure 7:**
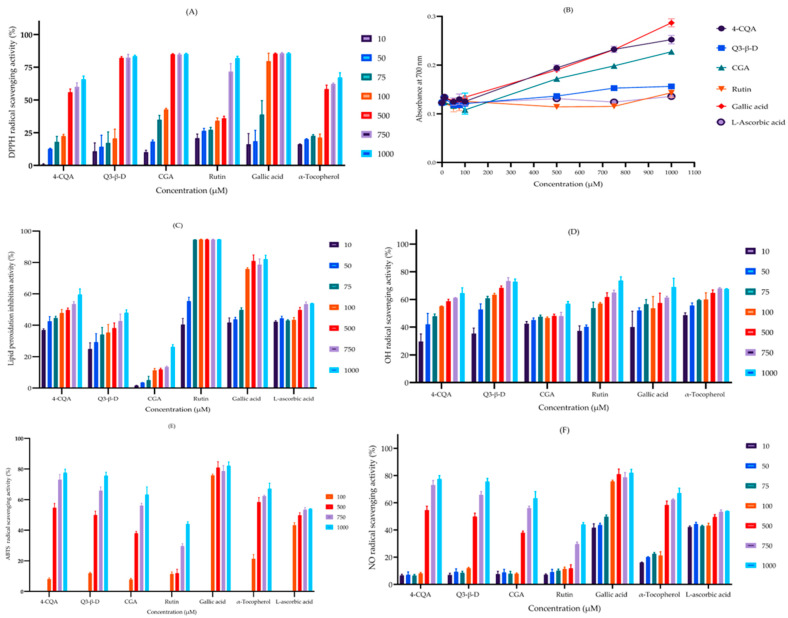
Assessment of phytochemical radical-scavenging and antioxidant abilities. Phytochemicals and antioxidants were assayed for their relative radical-scavenging and antioxidant activities using the (**A**) DPPH, 2,2-diphenyl-1-picrylhydrazyl radical scavenging assay; (**B**) ferric reducing antioxidant power (FRAP) activity assay; (**C**) lipid peroxidation inhibition activity assay (LPI); (**D**) hydroxyl (OH) radical-scavenging assay; (**E**) 2,2′-azino-bis-3-ethylbenzthiazoline-6-sulfonic acid (ABTS) radical cation-scavenging assay, and (**F**) nitric oxide (NO) radical-scavenging assay. Histograms represent the mean ± SEM of three replicate assays (n = 3). Abbreviations: 4-CQA, 4-*O*-caffeoylquinic acid; Q3-β-D, quercetin 3-β-D-glucoside; CGA, chlorogenic acid.

The ability of the phytochemicals (in the concentration range of 10‒1000 µM) to act as radical scavengers was assessed by measuring their ability to donate hydrogen atoms and neutralize 2,2-diphenyl-1-picrylhydrazyl (DPPH) free radicals. The relative potency of DPPH radical-scavenging activity was in the order of CGA > Q3-β-D > rutin > 4-CQA (Figure 7A). The effective drug concentration that produced a 50% effect (EC_50_) was calculated for each of the phytochemicals using non-linear regression and the values are shown in Table 3. When compared to the known antioxidant compound α-tocopherol, all the phytochemicals except 4-CQA displayed more potent antioxidant (radical-scavenging) capabilities, but they were all less potent than gallic acid (Figure 7A and Table 4).

#### 3.4.2. Ferric Reducing Antioxidant Power Assay

Relative phytochemical antioxidant activity was assessed using the ferric reducing antioxidant power (FRAP) assay. The phytochemicals all displayed comparable reducing activity in the order of 4-CQA > CGA > Q3-β-D > rutin (Figure 7B). In comparison, all the phytochemicals except rutin displayed more potent antioxidant activity than α-tocopherol but were marginally less potent than gallic acid (Table 4).

#### 3.4.3. Lipid Peroxidation Inhibition Assay

The phytochemicals were assessed using the lipid peroxidation inhibition (LPI) assay to determine their ability to limit ROS or free radical attack of lipids in cell membranes and the formation of lipid peroxides. Rutin was the most potent inhibitor of lipid peroxidation and 4-CQA also displayed high efficacy, with an EC_50_ value similar to L-ascorbic acid. Collectively, the order of potency for LPI was rutin > 4-CQA > Q3-β-D > CGA. Rutin and 4-CQA were more potent inhibitors than gallic acid (Figure 7C and Table 4).

#### 3.4.4. Hydroxyl Radical-Scavenging Assay

The ability of the phytochemicals to scavenge hydroxyl radicals (•OH) was assessed. Q3-β-D and α-tocopherol had similar activities and were the most potent radical scavengers. Rutin, 4-CQA, and gallic acid also showed strong radical-scavenging abilities. OH radical-scavenging activity was in the order of Q3-β-D > rutin > 4-CQA > CGA (Figure 7D and Table 4).

#### 3.4.5. 2,2′-Azino-bis(3-ethylbenzothiazoline-6-sulfonic acid) Radical-Scavenging Assay

An assessment of the phytochemicals’ ability to scavenge 2,2′-azino-bis(3-ethylbenzothiazoline-6-sulfonic acid) radical cations (ATBS•^+^) showed that the potency was in the order of 4-CQA > Q3-β-D > CGA > rutin. 4-CQA and Q3-β-D had similar efficacies as α-tocopherol but gallic acid was the most effective ABTS radical cation scavenger, with a relatively low EC_50_ (Figure 7E and Table 4).

#### 3.4.6. Nitric Oxide Radical-Scavenging Assay

The ability of the phytochemicals to neutralize the nitric oxide radical (•NO) was examined and all phytochemicals displayed moderate activity in the order of 4-CQA > Q3-β-D > CGA > rutin. Notably, 4-CQA and Q3-β-D exhibited relatively strong scavenging activity, whereas the antioxidants L-ascorbic acid and gallic acid were less effective and had higher EC_50_ values (Figure 7F and Table 4).

## 4. Discussion

*M. oleifera* produces a diverse array of secondary metabolites with nutraceutical potential [18,19,20]. Our initial analysis of the phytochemicals in aqueous and ethanolic extracts of *M. oleifera* using LC-MS/MS identified a variety of bioactive compounds, including some with well-documented antioxidant properties (Table 1). Four phytochemicals, 4-*O*-caffeoylquinic acid, quercetin 3-β-D-glucoside, chlorogenic acid, and rutin, were further analysed and found to possess weak acetyl- and butyrylcholinesterase inhibitory activities compared with the currently prescribed ChEI drugs rivastigmine, donepezil, and galantamine. Complementary in silico analyses of the phytochemicals as ligands for AChE and BuChE substantiated their proposed dual cholinesterase inhibitor activity. Additionally, these phytochemicals also displayed useful radical-scavenging and antioxidant capabilities that were often comparable and sometimes more potent than the known antioxidants gallic acid, α-tocopherol (vitamin E), and ascorbic acid (vitamin C). Hence, the combined dual ChEI and potent antioxidant activities indicate that these phytochemicals could be used to limit some of the pathological deficits experienced by patients with AD.

Our LC-MS/MS analysis of aqueous and ethanolic extracts of *M. oleifera* revealed the presence of diverse chemical groups, including phenolic compounds, flavonoids, lignans, proanthocyanidins, monoterpinoids, norisoprenoids, mono- and polyunsaturated fatty acids, saturated fatty acids, and amino acids (Table 1). The identification of these compounds aligns with other analytical studies of *M. oleifera*, which have considered a range of plant parts [18,19,20] and documented the presence of some of the phytochemicals detailed in Table 1.

Extracts of *M. oleifera* display neuroprotective and cognitive enhancement capabilities [24,25,26], and this prompted us to specifically investigate whether its phytochemicals could enhance or sustain cholinergic signalling via cholinesterase inhibitor activity. The tested phytochemicals 4-*O*-caffeoylquinic acid (4-CQA), quercetin 3-β-D-glucoside (Q3-β-D), chlorogenic acid (CGA), and rutin all exhibited varying degrees of (in vitro) inhibitory activity against both AChE and BuChE enzymes. The inhibitory potential against hAChE was in the order of 4-CQA > Q3-β-D > CGA > rutin; this order was similar for eeAChE except Q3-β-D was more potent than 4-CQA. By comparison, for hBuChE, the phytochemicals were ranked in the order of rutin > 4-CQA > Q3-β-D > CGA and for equine serum BuChE, it was 4-CQA > Q3-β-D > rutin > CGA.

Caffeoylquinic acids (a family of compounds that are esters of caffeic acid with quinic acid), such as 4-*O*-caffeoylquinic acid (4-CQA), are phytochemicals that are often obtained through the consumption of fruits and vegetables as well as coffee, which have recognized anti-inflammatory and antioxidant properties [53]. Our study shows that, for the phytochemicals tested, 4-CQA displayed weak cholinesterase inhibitor activity but was the strongest inhibitor of hAChE and second-best inhibitor of hBuChE.

Flavonoids, such as quercetin 3-β-D-glucoside (Q3-β-D), constitute a broad chemical group, and are often obtained through the dietary intake of fruits and vegetables, as well as beverages, and have purported antioxidant and nutraceutical properties [54]. An assessment of the AChE inhibitory activity of members of the flavonoid class revealed that quercetin had weak but detectable AChE and BuChE inhibitor, with an IC_50_ of approximately 20 µM, approximately 200 times higher than that of tacrine, a compound that had been employed as a clinical ChEI [55,56,57]. Our studies using Q3-β-D (also known as isoquercetin), which has a similar structure to quercetin, showed similarly weak AChE and BuChE inhibitory activities, with IC_50_ values of just below 1 mM (Table 2).

Chlorogenic acid (CGA) (5-caffeoylquinic acid) is a dietary polyphenol and member of the caffeoylquinic acid family. It is structurally similar to 4-CQA and has diverse biologically active properties that include anti-inflammatory, antioxidant, as well as metabolic homeostasis modulation activities [58]. Our data suggests that CGA can act as a weak ChEI, and this concurs with other studies that have reported inhibitory activity against hAChE (IC_50_ of 0.41 mM) [59].

Rutin, also known as quercetin-3-rutinoside, is a flavonoid found in a number of dietary sources including teas and fruits and has a range of pharmacological and nutraceutical properties [60,61]. Rutin displayed weak dual ChEI activities (low mM IC_50_s) and was the most potent inhibitor of hBuChE. Similarly, studies performed using rat brain homogenates reported that rutin was capable of dual-cholinesterase inhibition in vitro, with AChE and BuChE IC_50_ values of 0.219 and 0.288 mM, respectively [62].

To provide further insight into the potential ChEI activity of the phytochemicals and support for our in vitro findings, molecular docking was performed to study the binding interactions of each of the phytochemicals with the active site residues of human AChE and BuChE. The docking simulations suggested that these phytochemicals interacted with critical active site residues. This provided a quantitative determination of their relative binding affinities and potential inhibitory mechanisms. Rutin displayed the highest binding affinities for both hAChE and hBuChE, with binding energies of −14.81 kcal/mol and −12.03 kcal/mol, respectively. Its ability to form extensive hydrogen bonds with catalytic and peripheral residues, combined with π-π stacking interactions, suggested a strong capacity to obstruct substrate access and stabilize its position within the active sites of these enzymes. However, in contrast to the relatively high affinity proposed by computational predictions, rutin was not the most efficient in vitro inhibitor (Table 2), although this could reflect the relatively strong predicted interaction, which could result in slow off-binding kinetics and decreased inhibitory activity, but we did not investigate this further.

The other three phytochemicals, 4-CQA, Q3-β-D, and CGA, had similar and relatively strong predicted binding affinities to hAChE: −9.12, −9.96, and −9.69 kcal/mol, respectively; these affinities are stronger (more negative) than that of galantamine at −7.7 kcal/mol [63]. However, all four tested phytochemicals displayed relatively weak in vitro cholinesterase inhibitory capacity. This highlights the difference between the dynamic nature of enzymatic interactions in vitro from the static conditions typically modelled in docking studies, as in silico simulations typically assume optimal binding conformations and do not account for factors such as enzyme flexibility and induced conformational changes.

Clearly, even at the high purity examined, these phytochemicals have inferior in vitro potencies than the current FDA-approved drugs (rivastigmine, donepezil, and galantamine) or known inhibitors (eserine and ethopropazine for AChE and BuChE, respectively) that we examined under identical kinetic conditions. Hence, these phytochemicals had IC_50_ values in the high nanomolar to low micromolar range, consistent with other studies that reported their in vitro efficacy values [52,64,65]. Thus, these phytochemicals would be inappropriate as therapeutic interventions if applied as a monotherapy. Nevertheless, the restoration of depleted cholinergic signalling is only one element of AD pathology, so treatments that address additional components of disease pathophysiology, such as redox stress, will likely have enhanced therapeutic utility. Therefore, we also evaluated the antioxidant properties of these phytochemicals using a panel of in vitro assays (Figure 7). The phytochemicals (4-CQA, Q3-β-D, CGA, and rutin) exhibited varying degrees of antioxidant activity, with a demonstrated ability to scavenge free radicals. 4-CQA was the most potent antioxidant in terms of performance across the panel of assays and was the most potent for three assays: FRAP, ATBS•^+^, and •NO. Interestingly, each of the other phytochemicals was the most powerful antioxidant in one other assay: Q3-β-D for •OH scavenging, CGA for DPPH• scavenging, and rutin for the LPI assay (Table 4). Other independent studies have also documented strong antioxidant activities for chlorogenic acid and rutin [38,44,45]. In addition, a panel of antioxidant assays performed using rutin generated IC_50_ values of approximately 184–938 µM [62], comparable to our results.

Despite the relatively weak ChEI activity of phytochemicals in vitro, extracts of *M. olefiera* have been utilized as a neuroprotective agent in vivo [22,23,24,25]. This likely reflects the effects of multiple and potentially interacting phytochemicals rather than individual agents, and these could collectively contribute to ChEI activity as well as additional mechanisms to mitigate redox stress and other AD-related pathologies, such as the accumulation of Aβ [66]. Indeed, our data indicates that the phytochemicals were each superior in one (or more) antioxidant assay. However, typically, pure chemical entities are used for randomized control trials (RCTs), such as chlorogenic acid or α-tocopherol [16,58,67,68], although several human trials with *M. olefiera* extracts have been undertaken to evaluate its safety and efficacy profile [69]. To date, the relative levels each of the phytochemicals within an *M. olefiera* extract and their contribution to its collective efficacy are unknown, but our future studies will aim to address this through more in-depth quantitative studies of *M. olefiera* extracts.

In summary, the findings of the current study provide evidence that phytochemicals derived from *M. oleifera* have weak dual ChEI activity and potent antioxidant capabilities. This supports the potential use of *M. olefiera* phytochemicals and/or extracts to enhance cognitive performance and as a possible means to combat some of the pathophysiological elements that arise in AD. Dietary intake or supplementation with *M. olefiera* (or its phytochemicals) could provide a beneficial source of nutraceuticals with sustained low dosing; it also has potential as a treatment or prophylaxis to stave off the development of AD.

## 5. Conclusions

Extracts of the *M. oleifera* plant contain a range of phytochemicals and secondary metabolites that are also commonly encountered in fruits, vegetables, and beverages such as teas and coffee. Our data show that phytochemicals from *M. oleifera* could have nutraceutical properties arising from their ChEI and antioxidant activities that could be beneficial as a natural treatment and/or chronic dietary supplement that can treat and limit the development or progression of neurodegenerative diseases such as AD. However, more in vivo studies and RCTs are needed to validate this proposition and confirm the phytochemicals’ safety and efficacy, as well as their potential bioavailability, pharmacokinetics, and pharmacodynamics limitations.

## Figures and Tables

**Table 1:** LC-MS results of *M. oleifera* extracts and compound antioxidant activities.

Name of Compound	Formula	Exact Mass	*Moringa oleifera* Plant Extracts		Antioxidant Activity	Reference(s)
			PBS	Ethanol		
**Phenolic compounds and phenolic acids**						
Protocatechuic acid	C_7_H_6_O_4_	154.0268	Detected	Detected	✓	[31]
Syringic acid	C_9_H_10_O_5_	198.0530	Detected	Detected	✓	[32]
Vanillic acid	C_8_H_8_O_4_	168.0425	Detected	Detected	✓	[33]
Gallic acid	C_7_H_6_O_5_	170.0217	Detected	Not Detected	✓	[34,35,36]
4-Hydroxybenzoic acid	C_7_H_6_O_3_	138.0319	Detected	Detected	✓	[36,37]
Benzoic acid	C_7_H_6_O_2_	122.0371	Detected	Not Detected		
2,5-Dihydroxybenzoic acid	C_7_H_6_O_4_	154.0268	Detected	Detected	✓	[36,37]
Chlorogenic acid	C_16_H_18_O_9_	354.0951	Detected	Detected	✓	[38]
Methyl 4-caffeoylquinic acid	C_17_H_20_O_9_	368.1108	Detected	Detected	-	
**Flavonoids**						
Dihydrokaempferol	C_15_H_12_O_6_	288.0634	Detected	Detected	-	
Luteolin	C_15_H_10_O_6_	286.0478	Not Detected	Detected	✓	[39]
Quercetin	C_15_H_10_O_7_	302.0426	Not Detected	Detected	✓	[40,41,42]
Taxifolin deoxyhexose or taxifolin	C_15_H_12_O_7_	304.0584	Detected	Detected	✓	[43]
Quercetin-3-O- rutinoside (rutin)	C_27_H_30_O_16_	610.1519	Not Detected	Detected	✓	[44,45]
Quercetin 3-O-glucoside (isoquercitrin)	C_21_H_20_O_12_	464.0950	Not Detected	Detected	-	
Kaempferol rhamnoside	C_21_H_20_O_10_	432.1048	Detected	Not Detected	-	
Isoorientin	C_21_H_20_O_11_	448.0996	Not Detected	Detected	-	
**Lignans**						
(+)-Isolariciresinol	C_20_H_24_O_6_	360.1575	Detected	Detected	✓	[46]
(+)-lariciresinol	C_20_H_24_O_6_	360.1575	Detected	Detected	✓	[47]
Dihydroconiferyl alcohol	C_10_H_14_O_3_	182.0945	Detected	Detected	-	
**Proanthocyanidins**						
(+)-Catechin	C_15_H_14_O_6_	290.0785	Not Detected	Detected	✓	[39,48]
**Monoterpenoids**						
(+)-Menthiafolic acid	C_10_H_16_O_3_	184.1100	Detected	Not Detected	-	
(E,Z)−2,6-dimethyl−2,6-octadiene−1,8-diol	C_10_H_18_O_2_	170.1309	Not Detected	Detected	-	
**Norisoprenoids**						
(−)-Loliolide	C_11_H_16_O_3_	196.1099	Not Detected	Detected	✓	[49]
**Major fatty acids**						
**Monounsaturated fatty acids**						
Oleic acid	C_18_H_34_O_2_	282.2558	Detected	Detected	-	
Palmitoleic acid	C_16_H_30_O_2_	254.2247	Not Detected	Detected	-	
**Polyunsaturated fatty acids**						
Linoleic acid	C_18_H_32_O_2_	280.2401	Not Detected	Detected	-	
Linolenic acid	C_18_H_30_O_2_	278.2245	Not Detected	Detected	-	
**Saturated fatty acids**						
Palmitic acid	C_16_H_32_O_2_	256.2403	Not Detected	Detected	-	
Stearic acid	C_18_H_36_O_2_	284.2714	Not Detected	Detected	-	
**Amino acids**						
Alanine	C_3_H_7_NO_2_	89.0477	Detected	Detected	-	
Lysine	C_6_H_14_N_2_O_2_	146.1055	Detected	Not Detected	-	
Arginine	C_6_H_14_N_4_O_2_	174.1116	Not Detected	Detected	-	
Methionine	C_5_H_11_NO_2_S	149.0511	Detected	Not Detected	-	
Phenylalanine	C_9_H_11_NO_2_	165.0790	Detected	Detected	-	
Proline	C_5_H_9_NO_2_	115.0632	Detected	Detected	-	
Glutamic acid	C_5_H_9_NO_4_	147.0532	Detected	Detected	-	
Serine	C_3_H_7_NO_3_	105.0426	Detected	Detected	-	
Glycine	C_2_H_5_NO_2_	75.0320	Detected	Detected	-	
Threonine	C_4_H_9_NO_3_	119.0582	Detected	Detected	-	
Histidine	C_6_H_9_N_3_O_2_	155.0694	Detected	Detected	-	
Tryptophan	C_11_H_12_N_2_O_2_	204.0901	Detected	Detected	-	
Tyrosine	C_9_H_11_NO_3_	181.0741	Detected	Detected	-	
Isoleucine	C_6_H_13_NO_2_	131.0946	Detected	Detected	-	
Valine	C_5_H_11_NO_2_	117.0788	Detected	Detected	-	
Leucine	C_6_H_13_NO_2_	131.0948	Not Detected	Detected	-	
**Other compounds**						
Cellotetraose	C_24_H_42_O_21_	666.2225	Not Detected	Detected	-	
Sucrose	C_12_H_22_O_11_	342.1155	Detected	Detected	-	
Quinic acid isomer 1	C_7_H_12_O_6_	192.0635	Detected	Detected	-	
Vitamin C	C_6_H_8_O_6_	176.0322	Detected	Detected	✓	[50]

**Table 2:** Comparison of inhibitory capacity of phytochemicals and known cholinesterase inhibitors.

Agent	Enzyme IC_50_ (µM)			
	hAChE	hBuChE	eeAChE	esBuChE
4-*O*-caffeoylquinic acid	782 ± 106	770 ± 116	688 ± 58	903 ± 139
Quercetin 3-β-D-glucoside	971 ± 157	931 ± 49	868 ± 74	4029 ± 388
Chlorogenic acid	1362 ± 151	2049 ± 234	1034 ± 137	12,389 ± 4512
Rutin	2677 ± 296	761.4 ± 106	759 ± 104	6734 ± 523
Rivastigmine	8.7 ± 13	3.6 ± 0.3	6.7 ± 1.6	3 × 10^−4^ ± 1 × 10^−4^
Donepezil	1.6 ± 0.5	561 ± 132	3.5 ± 1.1	13.8 ± 0.8
Galantamine	8.7 ± 2.2	334 ± 23	6.5 ± 0.7	0.1 ± 0.05
Eserine	2 ± 0.4	-	0.1 ± 0.02	-
Ethopropazine	**-**	13.8 ± 0.8	**-**	1 × 10^−2^ ± 3 × 10^−4^

Values represent the mean ± SEM of the actual or estimated IC_50_ values from at least three experiment trials (n = 3), as determined by non-linear regression. Abbreviations: hAChE, human acetylcholinesterase; hBuChE, human butyrylcholinesterase; eeAChE, *Electrophorus electricus* acetylcholinesterase; esBuChE, equine serum butyrylcholinesterase.

**Table 3:** The molecular docking interactions and binding affinities of the phytochemicals to human AChE and BuChE enzymes.

Phytochemical	hAChE Binding Energy (kcal/mol)	Key Interactions	hBuChE Binding Energy (kcal/mol)	Key Interactions
**4-CQA**	−9.12	Arg296 (H bond), Tyr341 (π interaction), HOH 712 (H bond)	−7.97	Glu197, Asp70 (H bond), Pro285, Tyr128 (H bond), Tyr332 (π interaction)
**Q3-β-D**	−9.96	Glu202 (H bond), Trp86 (π interaction), Tyr337 (π-π stacking), water (H bond)	−10.27	Pro285, Trp82, Glu197 (H bond), Tyr332 (π interaction)
**CGA**	−9.69	Arg296 (H bond), Trp86, Tyr337 (H-π interaction)	−9.69	Glu197, Trp82 (H bond), Tyr332 (π interaction)
**Rutin**	−14.81	Glu202, Asp74 (H bond), Tyr72, Arg296 (H bond), Trp86 (π-π stacking)	−12.03	Glu197, Pro285, Trp82, Asn68, Asp70 (H bond), Tyr332 (π stacking)

**Table 4:** EC_50_ values of selected phytochemicals from radical-scavenging and antioxidant assays.

Agent	EC_50_ (µM)					
	DPPH•	FRAP	LPI	•OH	ATBS•^+^	•NO
4-CQA	431 ± 28	1287 ± 45	207 ± 63	120 ± 30	382 ± 44	125 ± 30
Q3-β-D	224 ± 27	1785 ± 107	673 ± 165	55 ± 12	436 ± 32	261 ± 77
CGA	141 ± 8	1450 ± 54	3332 ± 319	231 ± 80	673 ± 44	383 ± 99
Rutin	305 ± 55	1941 ± 131	26 ± 3	90 ± 21	1717 ± 206	383 ± 87
Gallic acid	86 ± 15	1243 ± 49	372 ± 33	80 ± 23	53 ± 15	972 ± 107
α-Tocopherol	369 ± 34	1770 ± 100	-	53 ± 15	414 ± 22	368 ± 34
L-Ascorbic acid	-		182 ± 24	-	526 ± 110	720 ± 127

Abbreviations: ABTS•^+^, 2,2′-azino-bis(3-ethylbenzthiazoline-6-sulfonic acid) radical cation; 4-CQA, 4-*O*-caffeoylquinic acid; CGA, chlorogenic acid; DPPH•, 2,2-diphenyl-1-picrylhydrazyl radical; FRAP, ferric reducing antioxidant power; LPI, lipid peroxidation inhibition; •OH, hydroxyl radical; •NO, nitric oxide radical; Q3-β-D, quercetin 3-β-D-glucoside. -, not tested.

## Data Availability

The data used in the production of the figures is available on request from the first author.

## Supplementary Materials

The following supporting information can be downloaded at https://www.mdpi.com/article/10.3390/biomedicines13092148/s1, Supplementary Figure S1: Cholinesterase inhibition by FDA-approved drugs.

## Abbreviations

The following abbreviations are used in this manuscript:

4-CQA	4-*O*-caffeoylquinic acid
Q3-β-D	Quercetin 3-β-D-glucoside
CGA	Chlorogenic acid
