# Supplementary material for: Characterization of the Anticholinesterase and Antioxidant Properties of Phytochemicals from Moringa oleifera as a Potential Treatment for Alzheimer’s Disease
Source: Biomedicines. 2025 Sep 3;13(9):2148. doi: 10.3390/biomedicines13092148 (PMC12467246; doi:10.3390/biomedicines13092148)
Supplement: Supplementary file 1 [file biomedicines-13-02148-s001.zip › biomedicines-3812466-supplementary.pdf]

Supplementary Figure S1: Cholinesterase inhibition with FDA-approved drugs.

A

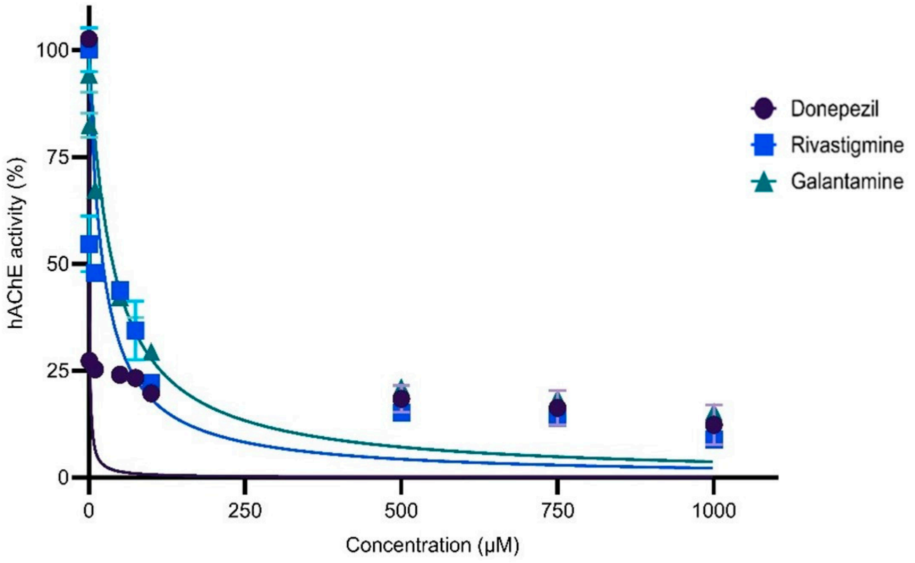

B

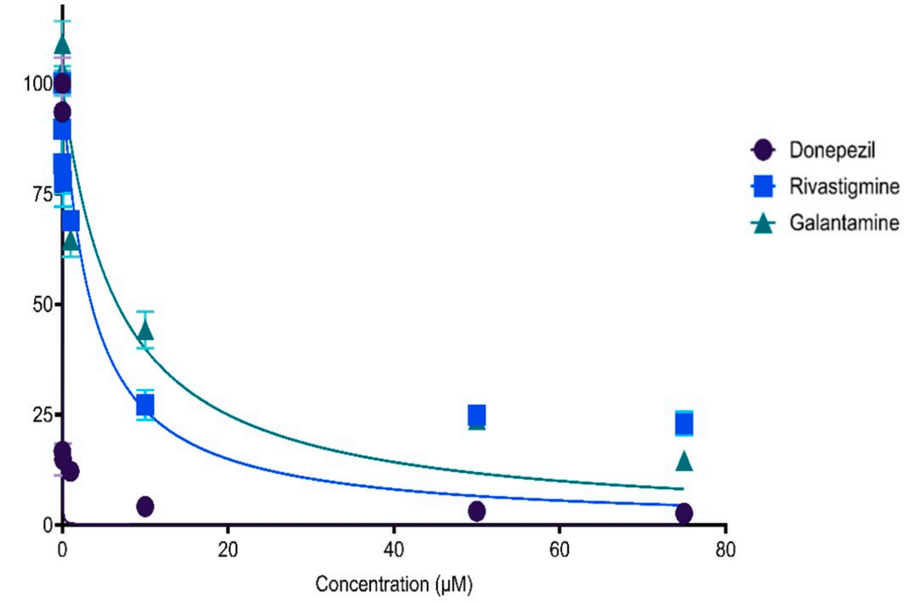

C

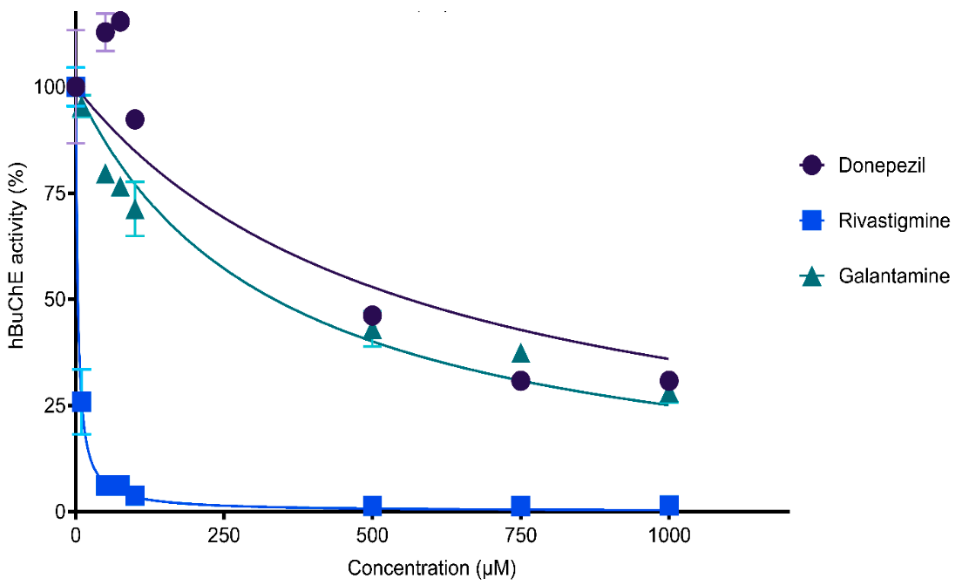

D

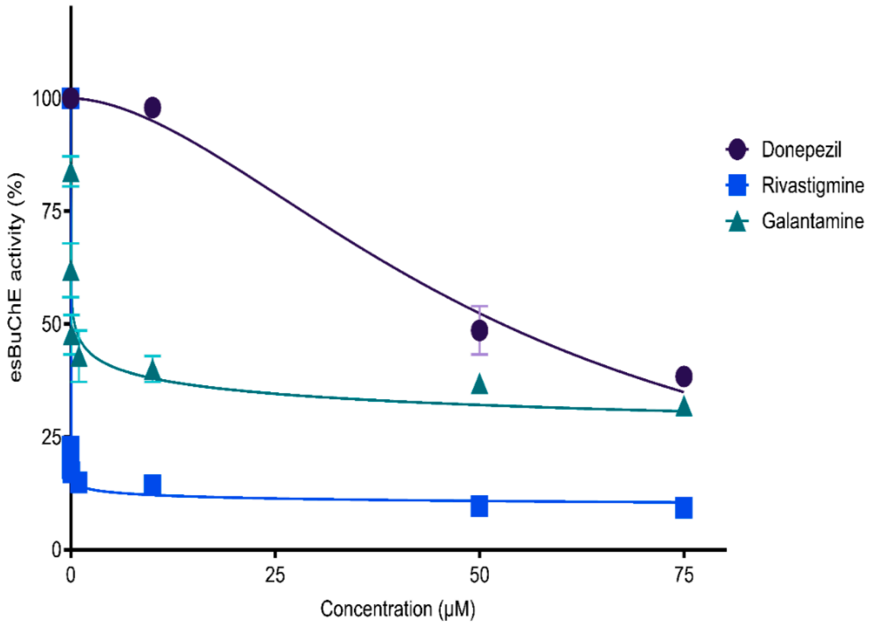

Cholinesterase inhibition assessed for (A) human acetylcholinesterase (hAChE), (B) *Electrophorus electricus* (electric eel) acetylcholinesterase (eeAChE), (C) human butyrylcholinesterase (hBuChE), and (D) equine serum butyrylcholinesterase (esBuChE)
